# Inhibition of IL-8-mediated endothelial adhesion, VSMCs proliferation and migration by siRNA-TMEM98 suggests TMEM98's emerging role in atherosclerosis

**DOI:** 10.18632/oncotarget.21408

**Published:** 2017-09-30

**Authors:** Guangxin Lv, Hongmei Zhu, Cai Li, Jingyu Wang, Dandan Zhao, Shuyao Li, Le Ma, Guohua Sun, Fang Li, Ying Zhao, Ying Gao

**Affiliations:** ^1^ Department of Biochemistry and Molecular Biology, Dalian Medical University, Dalian, 116044, China; ^2^ College of Stomatology, Dalian Medical University, Dalian, 116044, China; ^3^ Department of Clinical Laboratory, The First Affiliated Hospital of Dalian Medical University, Dalian, 116044, China; ^4^ Department of Immunology, Dalian Medical University, Dalian, 116044, China; ^5^ Liaoning Provincial Core Lab of Medical Molecular Biology, Dalian Medical University, Dalian, 116044, China

**Keywords:** atherosclerosis, IL-8, TMEM98, EC adhesion, VSMC proliferation and migration

## Abstract

Transmembrane protein 98 (TMEM98), known as a novel gene related to lung cancer, hepatocellular carcinoma, differentiation of T helper 1 cells and normal eye development, has no defined role reported in terms of atherosclerosis (AS). To investigate the potential involvement of TMEM98 during AS processes, its obvious secretion and expression has been initially characterized in hyperlipidemia patients’ serum and AS mice's serum respectively. We then explored the possible role of TMEM98 in the pathogenesis of AS *in vitro*. IL-8, a pro-atherogenesis cytokine, was used to induce the expression of TMEM98 in both endothelial cells (ECs) and vascular smooth muscle cells (VSMCs). Collectively, TMEM98 expression significantly increased in ECs and VSMCs, both induced by IL-8. Additionally, the adhesion ability of monocytes to ECs as well as the proliferation and migration of VSMCs were all decreased after siRNA-TMEM98 treatment. Furthermore, siRNA-TMEM98 dramatically inhibited the expression of ICAM-1 in ECs and the expression of p-AKT, p-GSK3β and Cyclin D1 from VSMCs, and AKT agonist partially restored the proliferation and migration of VSMC after siRNA-TMEM98 treatment. Taken together, siRNA-TMEM98 inhibits IL-8 mediated EC adhesion by down-regulating the expression of ICAM-1. Additionally, it also hinders the proliferation and migration of VSMCs through suppressing the AKT/GSK3β/Cyclin D1 signaling pathway. Our study provides sufficient evidence to support that TMEM98 could be a novel gene associated with AS for the first time.

## INTRODUCTION

Atherosclerosis (AS) causes clinical disease through luminal narrowing or by precipitating thrombi that obstruct blood flow to the heart (coronary heart disease), brain (ischemic stroke), or lower extremities (peripheral vascular disease). It is clearly a multifactorial disease following a number of different pathogenic mechanisms, with vast risk factors ranging from diabetes mellitus to various forms of high blood lipid profile [1–2]. It has becoming increasingly clear that AS is a maladaptive, non resolving chronic inflammatory disease over the past decades. There have been several inflammation mechanisms that can instigate the development, progression and complication of AS, among which, the atherosclerosis-related inflammatory cytokines that promote AS have been massively studied [3–6].

Interleukin-8 (IL-8) is a well-established pro-atherogenesis cytokine. There is sufficient evidence to support beyond any doubt of the involvement of IL-8 in the establishment and preservation of the inflammatory microenvironment of the insulted vascular wall of AS [7–9]. IL-8 can induce proliferation and chemotaxis of endothelial cells (ECs) and is an important chemoattractant for vascular smooth muscles cells (VSMCs). Nevertheless, little data are available in terms of the therapeutic potential of IL-8. Because of its crucial role in many aspects of immune mediated response, targeting IL-8 would produce many unwanted effect. More specifically, long-term blockage of non-specific chemotactic pathway might raise tolerability and safety concern especially for chronic condition like AS [10]. Thus, identification the molecular mechanisms that mediate IL-8-induced AS could reveal more appealing therapeutic targets for AS.

Transmembrane protein (TMEM) family is present in many cell types and mediates important physiological functions such as epidermal keratinization [11], cell autophagy [12], and smooth muscle contraction [13]. Among them, some members play a primordial role in inflammation. For example, TMEM9B is a lysosomal transmembrane protein and a key component of inflammatory signaling pathways [14]. Similarly, the effect of inflammation stimulation on mouse bone marrow-derived dendritic cells (BMDCs) using lipopolysaccharides (LPS) or poly-I: C stimuli results in down-regulation of TMEM176A and 176B transcription levels [15]. In this report, we describe the biological activity of novel molecule, Transmembrane protein 98 (TMEM98), to unveil its clinical significance and roles in AS. Little report is associated with human *TMEM98* gene. Its chromosome localization is in 17q11.2. There are two RNA splicing forms released from the NCBI database, TMEM98-v1 and TMEM98-v2 respectively [16]. Although there is slightly difference between them in the 5’ untranslated region, the coding products of them are almost the same, which consists of 226 amino acids, molecular weight of 24.6 kDa and isoelectric point of 4.718 [17]. TMEM98 was proposed as one of the signature genes for adenocarcinoma and also described as a novel chemoresistance-conferring gene in hepatocellular carcinoma. Recent study also highlighted its immune related property by discovering its modulatory effect on T helper (Th) 1 cells [18]. However, no review has been focused on recent advances as to whether the gene could involve in the AS progress.

In our present study, we strikingly found that significantly high secretion and expression of TMEM98 in hyperlipidemia patients’ serum as well as AS mice's serum and plagues. These findings demonstrated that TMEM98 may be a novel gene associated with AS and TMEM98 could participate in the development of AS. To test this hypothesis, we chose IL-8 with atherogenesis activity that promotes AS to induce the TMEM98 expression and attempted to explore the relevance of TMEM98 protein on IL-8-mediated adhesion of ECs as well as proliferation and migration of VSMCs. Moreover, knockdown of TMEM98 by small interference RNA (siRNA) was implemented to hamper this process, aiming to reveal the underlying mechanisms of TMEM98 on AS.

## RESULTS

### Secretion and expression of TMEM98 is increased in the serum and plaque of hyperlipidemia patients and AS mice

**Figure 1:**
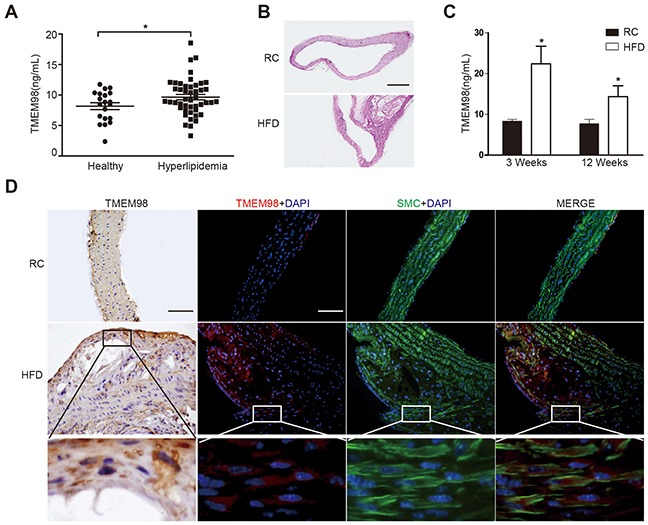
The secretion and expression of TMEM98 in serum of hyperlipidemia patients and in serum and plaque of AS mice TMEM98 secretion level in hyperlipidemia patients (n=46) serum and healthy donors (n=20) **(A)** HE staining was performed to show the morphology of blood vessels **(B)** TMEM98 level was examined by ELISA in HFD group serum (n=10) and RC group (n=10) **(C)** ^*^p<0.05 compared with the RC group. Representative immunohistochemistry and immunoflurescence images of TMEM98 in AS mouse model lesion. α-SMA staining marks smooth muscle cells, DAPI staining marks cell nuclei **(D)** HFD, high fat diet. RC, regular chow. Bars=200μm.

We found the secretion of TMEM98 protein in hyperlipidemia patients was higher than in healthy controls (paired 2-tailed t test, P < 0.05) (Figure 1A). The presence of hyperlipidemia predisposes the patient to AS through entry of LDL-C particles into arterial subendothelial spaces [19]. In order to identify the role of TMEM98, we established AS model by using ApoE^−/−^ mice fed with a high-fat diet as mouse models of AS have confirmed the proatherogenic properties of IL-8, associated with upregulation of ECs and activation of macrophage and vascular cell [6]. Figure 1B indicated that we successfully altered the arterial lumen and wall thickness to achieve locally obvious plaque formation. Serum and thoracic aortas were then obtained for detection of TMEM98 secretion (Figure 1C) and expression (Figure 1D) respectively. Interestingly, Figure 1C showed that TMEM98 secretion level in the HFD group was statistically higher than in the RC group in 3 week and 12 week accordingly. Consistent with the TMEM98 protein overexpression in atherosclerotic plaques by immunohistochemistry and immunofluorescence staining, the TMEM98 expression was also found to be mainly distributed in the smooth muscle cells from the blood vessel walls (Figure 1D).

### Expression of TMEM98 is increased in IL-8 treated blood vessel wall cell lines

**Figure 2:**
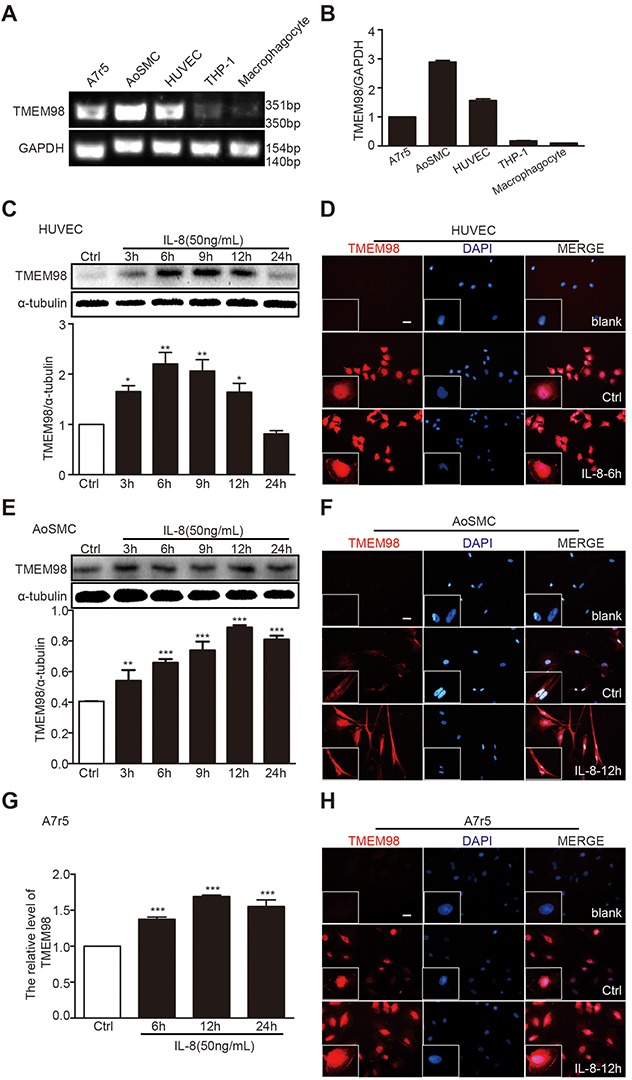
The expression of TMEM98 after treatment with IL-8 in HUVEC, AoSMC and A7r5 RT-PCR was performed to demonstrate TMEM98 expression in various cell lines **(A)** The graphic showed the statistical data of Figure 2A **(B)** HUVEC **(C and D)**, AoSMC **(E and F)**, and A7r5 cells **(G and H)** were treated with IL-8 at the indicated time lines (C-H). The cell lysates were analyzed by Western blotting using anti-TMEM98 antibody (C and E). mRNA level of *TMEM98* was analyzed by real time RT-PCR (G) Immunofluorescence staining confirmed the level and location of TMEM98 (D, F and H). The results were quantified and plotted in the bar graph and were expressed as the means ± SD (n=3). ^*^p<0.05, ^**^p<0.01, ^***^p<0.001 compared with the control group. Bars=100μm.

To further understand the possible impact of TMEM98 on AS, the next procedure was to assess the role of TMEM98 *in vitro*. We attempt to observe the expression of TMEM98 from vascular wall cells and AS-related cells treated by IL-8 to analyze whether TMEM98 gene expression is related to certain cell lines. The results indicated an apparent expression of TMEM98 mRNA in A7r5, AoSMC and HUVEC, but not in THP-1 and macrophagocyte (Figure 2A). Consequently, A7r5, AoSMC and HUVEC cell lines were nominated to observe the relative levels of TMEM98 protein expression over a time course after IL-8 stimulation. Within 3h to 24h, the TMEM98 protein expression in HUVEC started from 3 h till 12 h, with the peak at 6 h (Figure 2B) whereas the expression in AoSMC steadily increased as opposed to the control over 24 h (Figure 2D). We also observed that TMEM98 mRNA expression in A7r5 dramatically increased over 48h with the peak value at 12 h (Figure 2F).

Simultaneously, to further reveal and closely observe the distribution of TMEM98, immunofluorescence staining was employed into these three cell lines. All the data clearly demonstrated that TMEM98 expression in response to IL-8 was effectively enhanced in three different cell lines throughout the experiment period (Figure 2C, 2E and 2G), and depicted that TMEM98 protein is predominantly located in cytoplasm.

### siRNA-TMEM98 can decrease IL-8-mediated the adhesion of monocytes to ECs as well as the proliferation and migration of VSMCs

**Figure 3:**
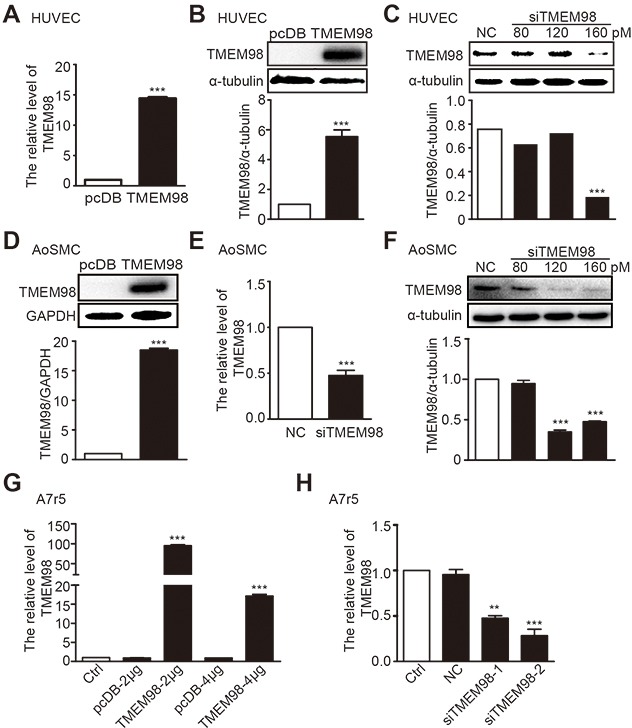
The expression of TMEM98 by down-, up-regulation *TMEM98* gene in HUVEC, AoSMC and A7r5 Real time RT-PCR was used to confirm the efficiency of siRNA-TMEM98 or TMEM98 plasmid in HUVEC, AoSMC and A7r5 **(A, E, G and H)**. Western blot assay was applied to confirm the efficiency of siRNA-TMEM98 or pcDB-TMEM98 plasmid in HUVEC and AoSMC **(B-D and F)**. pcDB represents vector of TMEM98 plasmid, TMEM98 represents pcDB-TMEM98 plasmid. NC represents negative siRNA as control, siTMEM98 represents siRNA-TMEM98. All results were mean±SD (n=3), ^**^p<0.01, ^***^p<0.001 compared with the control.

We examined the effect of TMEM98 on the functionality of blood vessel walls by using specific siRNA and plasmid that can reduce and increase the corresponding TMEM98 gene level in HUVEC, AoSMC and A7r5. As expected, after transfected with 4 μg/ml TMEM98 pcDNA3.1 plasmid into HUVEC, both mRNA and protein expression of TMEM98 significantly increased in sharp contrast with the same counterpart transfected with pcDNA3.1-myc-his (Figure 3A and 3B). With the concentration of 160 pM siRNA TMEM98 transfection, TMEM98 protein expression dropped obviously (Figure 3C). The similar trend can be observed with AoSMC cells (Figure 3D-3F). Additionally, in respond to different concentration of TMEM98 plasmid, real-time RT-PCR paradoxically reflects higher TMEM98 expression with 2 μg than 4 μg plasmid transfection in A7r5 (Figure 3G). While transfected with two different siRNA into A7r5 cells, the down-regulation of TMEM98 expression with siRNA-TMEM98-2 showed significantly higher than that with siRNA-TMEM98-1 (Figure 3H). Thus, the optimal effect of siRNA and plasmid on different cell lines was identified through the above experiments. The effects of TMEM98 on the functionality of blood vessel walls were then examined.

**Figure 4:**
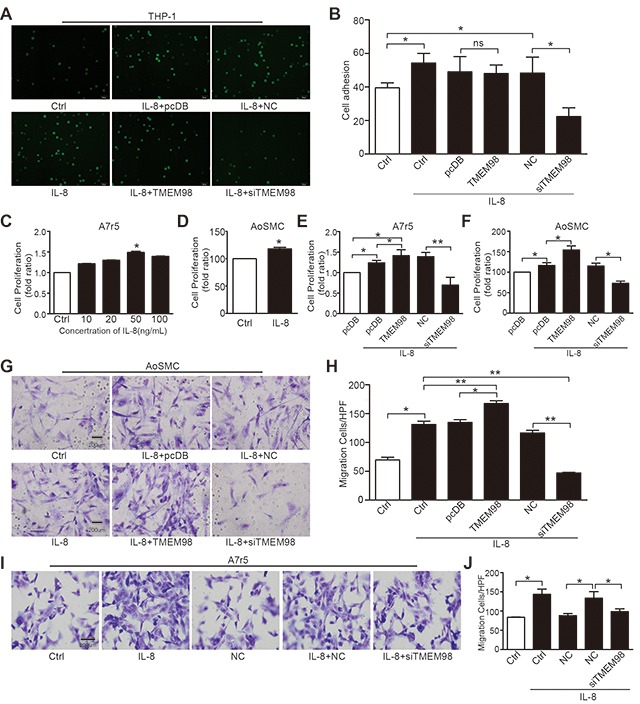
Effects of down-, up-regulation of *TMEM98* gene on IL-8-induced vascular wall cell function Cells after transfected with siRNA or plasmid of TMEM98 co-incubated with BCECF-labeled THP-1 cells for 30min in the presence of IL-8 **(A)** The number of adherent THP-1 cells in 9 random fields of view was counted. The graphic showed the statistical data of adhesion **(B)** Cell proliferation was measured by MTT assay **(C-F)**. A7r5 and AoSMC cells were dealt with IL-8 (C and D). A7r5 and AoSMC cells after transfected with siRNA or plasmid of TMEM98 were treatment with IL-8 (E and F). Boyden Chamber assay was conducted to investigate the migration of cells in AoSMC and A7r5 cells **(G–J)**. The graphic H and J showed the statistical data of migration in AoSMC and A7r5. The data were shown as the mean ± SD from 3 independent experiments. Significance was analyzed by using 1-way ANOVA. ^*^p<0.05, ^**^p<0.01, Bars=200μm.

IL-8 drives a pathogenic formation of AS through its multiple atherogenic effects, including monocyte adhesion to endothelium as well as proliferation and migration of VSMCs [7, 8]. Consistently, our results indicated that the cellular adhesion ability of monocytes to HUVEC significantly elevated after stimulation with 50 ng/mL IL-8 and remarkably decreased after siRNA-TMEM98 treatment. Notably, there was no increase of cellular adhesion to HUVEC with TMEM98 overexpression (Figure 4A and 4B). Regarding A7r5 and AoSMC, administration of 50 ng/ml IL-8 obviously promote the cell proliferation (Figure 4C and 4D) and migration (Figure 4G and 4I), and the opposite results were observed by using siRNA-TMEM98 (Figure 4E-4J). Interestingly, overexpressed TMEM98 indeed enhanced the effect on the proliferation and migration of AoSMC and (or) A7r5 (Figure 4E-4H). Results above revealed the TMEM98's effects on the progress of AS, in particular, siRNA-TMEM98 significantly down-regulates ECs adhesion and VSMCs proliferation and migration.

### siRNA-TMEM98 can inhibit IL-8 mediated ECs adhesion by down-regulating the expression of ICAM-1 and it also hinders the proliferation and migration of VSMCs through suppressing the AKT/GSK3β/Cyclin D1 signaling pathway

**Figure 5:**
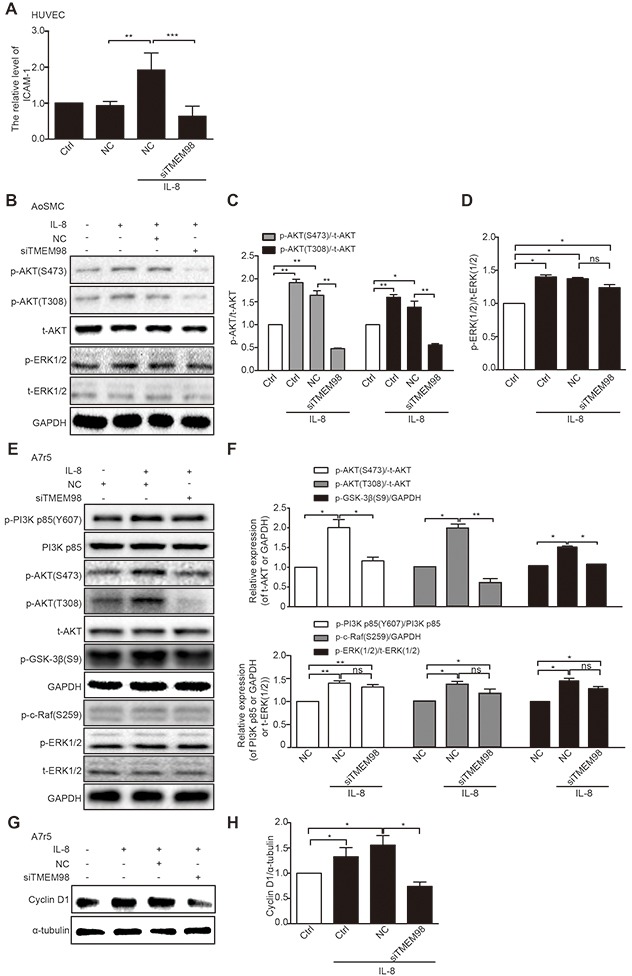
Investigation of possible signaling pathway related to TMEM98 in IL-8-treated vascular wall cells Real time RT-PCR analysis of the expression ICAM-1 after transfection of siRNA-TMEM98 into HUVEC cells **(A)** Western blot confirmed the protein level of AKT and EKR1/2 after transfection of siRNA-TMEM98 into AoSMC **(B)** The graphic showed the statistical data of Figure 5B **(C and D)**. Western blot confirmed the protein level of p-PI3K, PI3K p85 (Y607), p-AKT (S473), p-AKT (T308), t-AKT, p-GSK-3β, p-c-Raf (S259), p-ERK1/2 and t-ERK1/2 after transfection of siRNA-TMEM98 into A7r5 **(E)** The graphic showed the statistical data of Figure 5E **(F)** Cyclin D1 was examined by Western blot **(G and H)**. All experiments were performed in biologic triplicates. And the data were shown as the mean ± SD. ^*^p<0.05, ^**^p<0.01.

We investigated the possible mechanisms related to TMEM98's involvement in the regulation of vascular wall cells function. Real time RT-PCR was applied to detect the expression of EC adhesion related molecules ICAM-1, showing that IL-8 promoted the expression of ICAM-1 whereas siRNA-TMEM98 impaired the mRNA level of ICAM-1 in IL-8 mediated HUVEC adhesion (Figure 5A).

**Figure 6:**
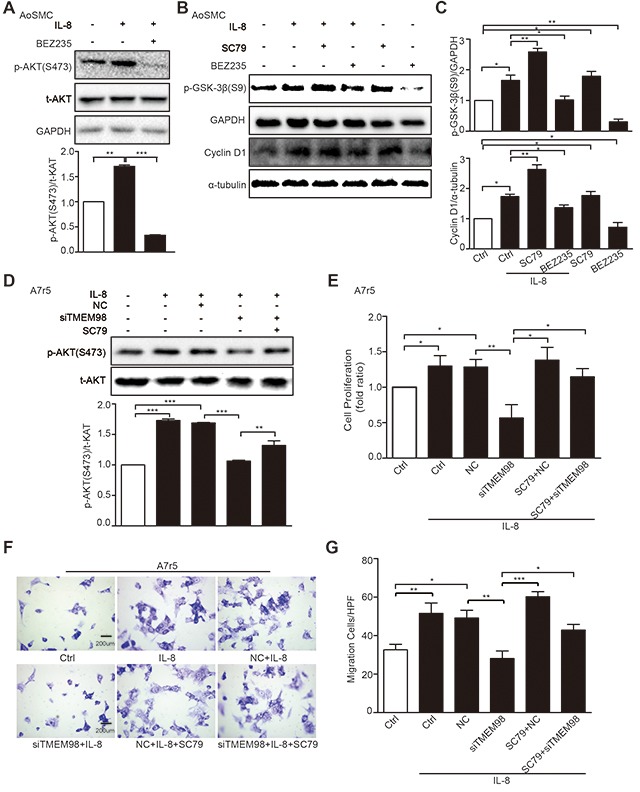
Effects of AKT signal pathway on IL-8-induced VSMC proliferation and migration with siRNA-TMEM98 treatment The activation status of p-AKT (S473) was detected after treatment with IL-8 and BEZ235 (PI3K inhibitor) by western blot **(A)**. The effects of SC79 and BEZ235 on the p-GSK-3β and Cyclin D1 in AoSMC cells by western blot **(B)**. The graphic showed the statistical data of Figure 6B **(C)**. Western blot was used to examine the activation status of p-AKT (S473) dealt with SC79 (AKT agonist) and (or) siRNA-TMEM98 in IL-8-treated A7r5 **(D)**. MTT and Boyden Chamber assay were assessed the proliferation and migration of IL-8-treated A7r5 cells dealt with SC79 and (or) siRNA-TMEM98 **(E-G)**. All results were mean ± SD (n=3). ^*^p<0.05, ^**^p<0.01, ^***^p<0.001 vs control.

We then tried to evaluate the effects of siRNA-TMEM98 on PI3K/AKT/GSK3β pathway that have been shown to control the progression of many VSMCs proliferation and migration [20, 21]. The results showed that p-AKT (T308 and S473) were potently reduced by siRNA-TMEM98 in IL-8-treated A7r5 and AoSMC (Figure 5B-5F). Furthermore, as PI3K and GSK-3β had been proved to be the upstream and downstream protein of AKT respectively, the activated status of the two proteins was then subsequently examined. The differential effect of TMEM98 on PI3K and GSK-3β receptor signaling is central to accounting for the effect on AKT signal pathway, p-PI3K was not changed whereas p-GSK-3β was markly down-regulated by siRNA-TMEM98 in IL-8-treated A7r5 cells (Figure 5E and 5F). IL-8 is therefore suspect not be able to induce an anti-inflammatory response in VSMCs lacking *TMEM98* gene. In agreement with this, we tried to use PI3K inhibitor (BEZ235) and AKT agonist (SC79) to confirm TMEM98's involvement in the proliferation and migration of VSMCs via AKT dependent signal pathway, finding that BEZ235 significantly decreased the p-AKT (S473), p-GSK3β and Cyclin D1 expression in IL-8-induced AoSMCs (Figure 6A-6C). On the contrary, SC79 dramatically increased the p-GSK3β and Cyclin D1 expression in IL-8-induced AoSMCs (Figure 6B and 6C). At the same time, it appeared higher protein level of p-AKT (S473) treated with SC79 compared with the siRNA-TMEM98 treatment group in IL-8-mediated A7r5 cells (Figure 6D). Notably, this effect was at least in part modulated *in vitro* by increased TMEM98 expression, as demonstrated by the fact that SC79 partially restored the proliferation and migration ability of VSMCs with siRNA-TMEM98 treatment (Figure 6E-6G).

We also examined the effects of siRNA-TMEM98 on the activity of ERK and expression of Cyclin D1. Activation of ERK signaling pathway was reported to mediate a range of cell fate decision, including cell proliferation, migration and transformation in VSMCs [22–25]. However, siRNA-TMEM98 did not markedly influence the activity of ERK1/2 in IL-8-treated AoSMC and A7r5 (Figure 5B-5F). And p-c-Raf from upstream protein ERK appeared no obvious change by siRNA-TMEM98 in IL-8-mediated A7r5 (Figure 5E and 5F). But siRNA-TMEM98 treatment did inhibit the expression Cyclin D1, a key regulator in the mammalian cell cycle, in IL-8-mediated A7r5 (Figure 5G and 5H).

## DISCUSSION

TMEM98 is discovered as a novel gene isolated from Human Genome Project (HGP) completed in 2005. So far, most of the knowledge regarding TMEM98 derived from the relevant prediction, which have been partially confirmed by experimental procedures in rather limited literature. TMEM98 is a type I membrane molecule by TMHMM analysis. By Signal P analysis, which consists of 21 amino acids on its N-terminal and implies that TMEM98 probably be a secretary protein. And Fu *et al* discovered an intense band at ~34kDa appeared in the supernatants from HEK293T cells transiently transfected with the pcDB-TMEM98 plasmid, which suggests that TMEM98 could possibly exist in secreted form [18].

BioGPS database analysis indicates that TMEM98 expression, located in nucleus and cytoplasm, was widely detected in most health tissues, especially with high expression in the olfactory bulb, fetal lung, thyroid gland, prostate, prefrontal cortex, the hypothalamus, smooth muscle cells and vascular endothelial cells (http://biogps.org). Awadalla and coworkers demonstrated that TMEM98 is also expressed in all eye tissues [17]. Pathologically, Mao *et al* discovered that TMEM98's expression in lung cancer patients’ tissues and their adjacent normal tissues [16]. Ng *et al*. confirmed that TMEM98 mRNA and protein level were significantly higher in tumor tissues of patients with hepatocellular carcinoma than in non-tumor tissues and healthy donor tissues [26], so does with TMEM98's high expression in adenocarcinoma [29].

Functionally, a few studies have investigated the TMEM98's involvement in several disease progressions. Recent findings have confirmed that in lung cancer cells, siRNA-TMEM98 significantly suppressed the invasion and migration of human A549 and H460 cells through down regulating MAT1, MMP-2, MMP-9 and RhoC [16]. Also, TMEM98 may confer chemoresistance of hepatocellular carcinoma through activation of the AKT pathway and deactivation of p53, suggesting its novel molecular target potential associated with intrinsic and acquired chemoresistance of hepatocellular carcinoma [26]. A missense mutation in this gene also resulted in Nanophthalmos 4 (NNO4) [17, 27, 28]. In adenocarcinoma, TMEM98's high expression leads to the worst prognosis of adenocarcinoma than other types of cancer [29]. Furthermore, TMEM98 could promote the differentiation of Th1 cells [18]. Be that as it may, no data suggest a possible association of acquired TMEM98 expression in the development of AS until now.

In our present *vivo* studies, TMEM98 protein level significantly increased in serum of hyperlipidemia patients and AS mice. Side by side comparison of TMEM98 expression conducted by immunohistochemical and immunofluorescence staining demonstrated that TMEM98 was largely expressed in the AS lesion compared with regular chow in ApoE^−/−^ mice, and was mainly distributed in the smooth muscle cells from blood vessel walls. When considered together with our results, the secretion and expression of TMEM98 were likely to have an effect on AS. As might be expected, we performed a series *in vitro* experiments to confirm the role of TMEM98 on AS.

AS is a chronic inflammatory process of the arterial wall driven by innate and adaptive immune responses. The innate response is instigated by the activation of both vascular cells and monocytes/macrophages. Subsequently, an adaptive immune response develops against an array of potential antigens presented to effector T lymphocytes by antigen-presenting cells. Vascular cells, ECs, and SMCs participate in the development of the disease by mediating leukocyte recruitment and vascular remodeling [4]. Importantly, in our *vitro* study, expression of TMEM98 appeared in ECs and VSMCs, but not in monocytes and macrophages. Therefore, in this report, we addressed the hypothesis that TMEM98 could promote an array of cellular processes that are essential for AS progression, including adhesion, proliferation and migration by using cultured ECs and VSMCs.

Inflammatory response of AS is primarily up-regulated by specific patterns of cytokine expression. There is sufficient evidence in the scientific literature to support beyond any doubt of the involvement of IL-8 in the establishment and preservation of the inflammatory microenvironment of the insulted vascular wall [10]. IL-8 stimulates AS development through various deleterious effects such as ECs dysfunction and inflammation, VSMCs proliferation and migration. Thus we chose IL-8 induced adhesion of ECs and proliferation and migration of VSMCs to evaluate whether TMEM98 is involved in the process of AS and its possible signaling pathway. It appeared that TMEM98 displayed dramatically higher expression in HUVECs and VSMCs when pretreated with IL-8, which is consistent with the previous report that TMEM98 is up-regulated by stimulation of inflammation [16]. On the other hand, ECs and VSMCs adhesion, migration and proliferation are very important components in AS. It has been proved that IL-8 could enhance the adhesion of ECs, and migration and proliferation of VSMCs. As a result, it is likely the promotion of ECs and VSMCs adhesion as well as proliferation and migration by IL-8 were mediated in part by an increase of TMEM98 expression. Consequently, we tested the TMEM98's contribution to the vascular dysfunction by gain- or loss- of TMEM98 expression dependent on IL-8 stimulation. Our data showed that siRNA-TMEM98 suppressed ECs adhesion as well as VSMCs proliferation and migration. Interestingly, overexpression of TMEM98 had significant enhanced effects on basal levels of proliferation and migration of VSMCs, but had no obviously effects on adhesion of ECs. Therefore, siRNA-TMEM98 would be expected to inhibit the cellular processes required for AS and it is necessary to further reveal the molecular mechanism of TMEM98 in IL-8-induced vascular wall dysfunction to explain this effect.

Binding and recruitment of circulating leukocytes to the vascular endothelium and further migration into subendothelium spaces are primary process in AS progress, which are mediated by a diverse group of cellular adhesion molecule like intercellular adhesion molecule 1 (ICAM-1) [30]. ICAM-1, as an important immunoglobulin like protein, is expressed on vascular EC surface [31], which linked to the firm adhesion of leukocytes to the apical surface of ECs and play a vital role during the inflammatory reaction and the immune response [32]. Except for the condition of endothelial barrier dysfunction, another functional alteration contributed to AS is the activation of the endothelium through proinflammatory mediators that control the vascular entry of leukocyte [30]. Proinflammatory cytokines, such as TNF-α, LPS can stimulate ECs and increase activation of ICAM-1 [30, 33], which in turn promote AS inflammation. Additional targets indispensable for the inflammatory activity of ICAM-1 may also involve in this effect, while data from our laboratory confirmed that down-regulation of TMEM98 in ECs induced by IL-8 inhibited the expression of adhesion molecule ICAM-1. Therefore, we speculate that ICAM-1 could involve in the adhesion of ECs mediated by TMEM98, and more in-depth research is still needed.

Given that some key signaling pathways, such as PI3K/AKT, MAPK/ERK1/2 and CyclinD1 pathways indispensable for cell survival, cell cycle transition, migration and metabolism [22–25, 34–36], we suspected whether there could be any degree of correlation occurring between TMEM98 functions and those above pathways. Our data showed that down-regulation of TMEM98 in VSMCs induced by IL-8 inhibited the expression of p-AKT, p-GSK-3β and Cyclin D1 respectively. On the contrary, levels of p-ERK1/2 and p-c-Raf remained unchanged in VSMCs induced by IL-8. Meanwhile, PI3K inhibitor and AKT agonist could suppress or elevate p-AKT, p-GSK3β and Cyclin D1 activities respectively. It is interesting to note that down regulation of p-AKT and p-GSK3β might be, at least in part, involved in siRNA-TMEM98-supressed proliferation and migration of VSMCs induced by IL-8, which was further approved by our results that AKT agonist partially restored the proliferation and migration ability of VSMCs with silence TMEM98. All pointed out that TMEM98 could up-regulate VSMCs biological behavior through the activation of the AKT/GSK3β/Cyclin D1 pathway, but not via ERK1/2 and c-Raf pathway. Nevertheless, as proliferation and migration of VSMCs is critical in the pathogenesis of AS, it is likely that the most effective extension of our current work will be to pinpoint other pivotal signaling pathways mediated by TMEM98.

**Figure 7:**
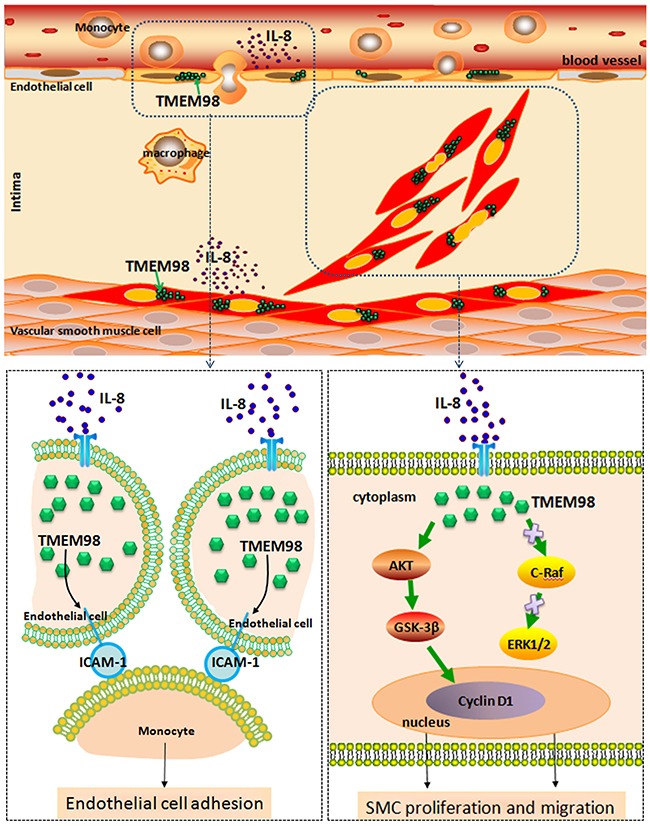
A schematic diagram of the TMEM98 expression in the blood vessel wall cells and its possible molecular pathways in terms of EC adhesion as well as VSMCs proliferation and migration

In summary, we functionally characterized TMEM98 and also indicated the relevance of this novel gene in the inflammation process of AS for the first time. Furthermore, down regulation of TMEM98 could inhibit monocytes adhesion into ECs through ICAM-1. siRNA-TMEM98 inhibited IL-8-induced VSMCs proliferation and migration partially through down regulating AKT/GSK3β/CyclinD1 (Figure 7), but not via alternative ERK and c-Raf pathway. Overall, our findings should provide the evidence that TMEM98 has future clinical promise in the development of novel therapeutic strategies, that is, broaden the application of TMEM98 as a potential target that can be used to treat IL-8-induced AS in future.

## MATERIALS AND METHODS

### Collection of human serum sample

A total of 66 participants (46 hyperlipidemia patients and 20 healthy) from the First Hospital of Dalian Medical University (Dalian, China) were enrolled in the study. The diagnosis of hyperlipidemia was based on total the level of cholesterol (TC), triglyceride (TG), low-density lipoprotein cholesterol (LDL-C) or high-density lipoprotein cholesterol (HDL-C) in serum. The blood samples were centrifuged at 3000g for 10min at 4°C. Written informed consent was obtained from all of the patients and the study were approved by and the institutional human ethical committee.

### Regents

Recombinant IL-8 was purchased from PeproTech, INC. Polyclonal antibodies against TMEM98 were purchased from Abcam. Alpha tubulin was purchased from Proteintech. Mouse anti-GAPDH monoclonal antibody was purchased from ZSGB-BIO. Monopoly antibodies against p-AKT (S473), p-AKT (T308), t-AKT, p-PI3K p85 (Y607), p-GSK3β (S9) and p-c-Raf (S259) were purchased from Cell Signaling Technology. The antibodies against p-ERK1/2, ERK and Cyclin D1 were purchased from Bioworld. The antibodies against of PI3K and p-PI3K were purchased from abcam. SC79 and BEZ235 were purchased from Selleck.cn. Fluorescein isothiocyanate (FITC)-conjugated anti-α-smooth muscle actin antibody (F3777) and phalloidin-tetramethyl rhodamine B isothiocyanate (P1951) were obtained from Sigma-Aldrich (St Louis, MO, USA).

### ELISA for TMEM98

TMEM98 levels in the serum were measured by Enzyme-linked Immunosorbnent Assay (ELISA) (Lengton Bioscience Co, Shanghai). Serum samples were placed in assay plate and incubated at 37°C for 30min. After five washes, samples were added horseradish peroxidase (HRP)-Conjugate Reagent and incubated at 37°C or 30min. Finally after another five washes, samples were incubated with Chromogen Solution A and B at 37°C for 10min and then the reactions were read at 490 nm in a microplate reader.

### Animal experiments

This study was conducted in accordance with the Guide for the Care and Use of Laboratory Animals published by the US National Institutes of Health (8th edition, 2011). ApoE^−/−^ mice were originally purchased from Beijing Vital River Laboratory Animal Technology Co., Ltd. These mice were randomly divided into the RC group (n=10, fed regular chow) and the HFD group (n=10). And AS model group that is HFD group were fed with a high fat diet consisting of 78% regular chow, 10% lard oil, 10% yolk powder, 1% cholesterol, and 0.2% bile salt from pig. After 12 weeks in the AS model, mouse aortas were excised. Whole blood samples were collected through aorta and kept in room temperature for 30min, then centrifuged at 2,700g for 10min. Sera were collected and stored at −80°C used for TMEM98 analysis. Samples of the aortic root were fixed in 4% paraformaldehyde and embedded in paraffin using standard procedures for subsequent immunohistochemical and immunofluorescence staining analyses. The animal protocol was approved by the local research ethics review board of the Animal Ethics Committee of Dalian Medical University.

### Immunohistochemical and immunofluorescence staining for tissues

The aortas were processed into 4μm thick serial sections and stained with hematoxylin/eosin to detect the development of atherosclerotic lesions. Additional aortic sections were used for immunohistochemical staining to detect TMEM98 or immunofluorescence staining for TMEM98, α -smooth muscle actin. Briefly, Paraffin sections were deparaffinized with dimethylbenzene and ethanol. Antigen was retrieved by incubation in 0.01 M citrate buffer (pH 6.0) at 95°C for 20min. The samples were rinsed three times with PBS, incubated for 15min at room temperature with a protein-blocking solution of 5% fetal bovine serum (FBS) in PBS (pH 7.5), washed three times with PBS, and then incubated with the primary antibodies (TMEM98 antibody, 1:50; FITC-conjugated α -smooth muscle actin antibody, 1:100) at 4°C overnight. The samples were then rinsed three times with PBS and incubated with the biotinylated secondary antibody or fluorochrome-conjugated secondary antibody for 40min at 37°C. Finally, the specimens were stained with DAPI. Digital images were taken using a fluorescence microscope (BX-51, TR32000, Olympus, Tokyo, Japan) with a charge-coupled device system.

### Cell culture

The A7r5 cell line, originally derived from an embryonic rat aorta, was purchased from American Type Culture Collection (Manassas, VA, USA). Cells were cultured in Dulbecco's modified Eagle's medium (DMEM; Gibco, Langley, OK, USA) supplemented with 10% fetal bovine serum (Gibco, Langley, OK, USA) at 37°C in a humidified atmosphere with 5% CO_2_ (CO_2_ incubator). Cells were grown to 80–90% confluence, and quiescence was achieved by serum starvation overnight. The AoSMC cell line was cultured in DMEM supplemented with 10% FBS, hEGF (0.5 ng/mL), hFGF (2 ng/mL) and insulin (5 μg/mL) at 37°C in a humidified atmosphere with 5% CO_2_. The human umbilical vein endothelial cells (HUVECs) line was cultured in DMEM supplemented with 10% FBS, hepes (3.4 g/L) (Amresco, USA) at 37°C in a humidified atmosphere with 5% CO_2_. The THP-1 cell line was cultured in RPMI 1640 medium containing 10% FBS at 37°C in a humidified atmosphere with 5% CO_2_. THP-1 differentiation into macrophages was induced by phorbol ester for 24 h.

### Cell staining for immunofluorescence microscopy

Immunofluorescence staining was performed on cultured cells. Treated HUVEC, AoSMC and A7r5 were fixed with 4% paraformaldehyde for 20min. After fixation, the cells were permeabilized with 0.1% TritonX-100 for 10 min, blocked with 5% normal goat serum or rabbit serum for 20min at room temperature and incubated with anti-TMEM98 antibody (1:50) overnight at 4°C. Then cells were rinsed in 0.1 mol/L phosphate buffered saline three times, incubated with corresponding secondary antibody (1:200) for 1h at 37°C and evaluated by fluorescence microscope (BX-51, TR32000, Olympus, Tokyo, Japan) with a charge-coupled device system. For negative controls, cells were incubated without secondary antibody.

### Adhesion of leukocytes to HUVECs

For adhesion of leukocytes to HUVECs, THP-1 monocytes were cultured in RPMI 1640 medium containing 10 μmol/L bis-carboxyethyl-carboxyfluorescein (BCECF; Beyotime Biotechnology). The BCECF-labeled THP-1 cells (4×10^5^ cells/mL) were incubated with HUVECs which were transfected with TMEM98 plasmid or siRNA for 1h. The adherent monocytes from 9 random selected vision areas were counted using fluorescence microscopy (Leica, Germany).

### Boyden chamber assay

Cell migration was assessed using the Boyden chamber assay (Neuro Probe, Inc., Gaithersburg, MD, USA), as previously described by Li *et al* [37]. In all experiments, collagen-coated polycarbonate filters with an 8 μm pore size were used. AoSMC and A7r5 cells were suspended in DMEM containing 0.4% BSA (DMEM–BSA) and loaded into upper wells (1×10^4^ cells/well) of the chamber. Lower wells were filled with DMEM-BSA containing 50ng/mL IL-8 or vehicle. After 5h incubation at 37°C, cells that had migrated to the lower surface of the filter were fixed in methanol, stained with Giemsa solution and observed under a light microscope. All conditions were tested in quadruplicate and the cell number from a randomly chosen high-power field (HPF, ×200 magnification) was counted in each well. In certain experiments, cells were pretreated with TMEM98 siRNA for 24h. The migration assay was performed in at least three independent experiments.

### MTT assay

To evaluate cell viability, 5×10^3^ cells were plated in each well of a flat-bottom 96-well culture plate, incubated in 100 μL DMEM medium for 24h and treated according to the described experimental conditions. After treatment, methyl thiazolyl tetrazolium (MTT) was added to each well to a final concentration of 0.5 mg/mL and incubated for 4h at 37°C in a humidified incubator containing 5% CO_2_. To dissolve the cells, we used 100 μL DMSO and measured the absorbance of the resulting solutions at 570 nm using a microplate reader (Thermo Scientific, USA). The results were presented as a percentage of cell viability (the optical density [OD] of the experiment samples/the OD of control group).

### Real-time PCR and RT-PCR analysis

RNA was prepared from cells, with TRIzol reagent (Invitrogen, Carlsbad, CA). For the quantification of mRNA, 1 μg of total RNA was reverse transcribed into cDNA using the TransScript One-Step gDNA Removal and cDNA Synthesis SuperMix (TransGen Biotech, Beijing). The cDNA was then subjected to quantitative PCR using the Mx3000p Multiplex Quantitative PCR System (Stratagene, Agilent, USA). The results were analyzed with Stratagene Mx3000 software and normalized to glyceraldehyde-3-phosphate dehydrogenase (GAPDH) as internal control. cDNA was amplified in a total volume of 50 μL using the TransGen 2 × EasyTaq PCR SuperMix Kit (TransGen Biotech, Beijing). Then equal amounts of PCR products were electrophoresed on 1% agarose gels and visualized by ethidium bromide staining. The specific bands of PCR products were analyzed by ImageJ Launcher system with GAPDH as an internal control for normalization. RT-PCR was performed in triplicates independently. The specific primers were listed in Table 1.

**Table 1:** Primer Sequence

*Gene*	*Forward primer(5′ to 3′)*	*Reverse primer(5′ to 3′)*	PCR size
***r-TMEM98***	TAGGTGTGCTGGCCACCATT	GACACTTGCCGACGTCTTCA	350
***h-TMEM98***	CTCATGTCCCACTGCATTGC	AGCTAGGGCTGCTTCTCGAA	351
***r-TMEM98-q***	CATCATCGTGGTGGCCAAA	GCAGGCATTCCTGGTCACTAG	151
***r-GAPDH***	GGAAGAATGGGAGTTGCTGTTGAAG	AATGGGGTGATGCTGGTGCTGAGTA	623
***r-GAPDH-q***	GCAAGTTCAACGGCACAG	GCCAGTAGACTCCACGACAT	140
***h-ICAM-1***	CAAGGTGACCGTGAATGTGC	CGTGGCTTGTGTGTTCGGTT	187
***h-GAPDH-q***	GAGTCAACGGATTTGGTCGT	GACAAGCTTCCCGTTCTCAG	185

### Western blotting

Cell lysates were prepared in RIPA lysis buffer containing 50mM Tris-HCl (pH 7.4), 150mM NaCl, 1% Triton X-100, 1% sodium deoxycholate, 0.1% SDS, 1 mM PMSF and a protease inhibitor cocktail. Protein samples were loaded and separated on a 10% SDS-polyacrylamide gel and transferred to a polyvinylidenedifluoride (PVDF) membrane (Millipore, Billerica, MA, USA). Membranes were washed with Tris-buffered saline (TBS) and blocked with 5% (w/v) non-fat milk in TBS-Tween20 (1%, v/v, TBST) for 1h at room temperature. After three washes of 5min each with TBST, membranes were incubated with primary antibodies (1:1000) in TBST with 5% (w/v) bovine serum albumin (BSA) overnight at 4°C. After three washes with TBST, the membranes were incubated with horseradish peroxidase-linked secondary antibodies (1:5000) for 1h at room temperature. Bands were detected by use of an enhanced chemiluminesence (ECL) detection kit (Thermo Electron Corp., Rockford, USA). The relative quantity of proteins was analyzed by use of using Image Gauge® Ver. 4.0 software (Fuji Film, Tokyo, Japan) and normalized to that of loading controls.

### Transfection of siRNAs and plasmids

Sequences of TMEM98 siRNA and negative control siRNA were designed and synthesized by GenePharma Bio Incorporated (USA). Human TMEM98 siRNA sequences were: 5′-CUUUCUGGCUUCGUUUGCATT-3′ (sense) and 5′-UGCAAACGAAGCCAGAAAGTT-3′ (antisense). Rat TMEM98 siRNA-1 sequences were: 5′-GCUAUGAUUCCAAGCCUAUTT-3′ (sense) and 5′-AUAGGCUUGGAAUCAUAGCTT-3′ (anti-sense). Rat TMEM98 siRNA-2 sequences were: 5′- GGUGCUAGUGACCAGGAAUTT-3′ (sense) and 5′- AUUCCUGGUCACUAGCACCTT-3′ (antisense). The negative control siRNA sequences were: 5′- |UUCUCCGAACGUGUCACGUTT-3′ (sense) and 5′- ACGUGACACGUUCGGAGAATT-3′ (antisense). Human TMEM98 (pcDB-TMEM98-myc-his) plasmid was given as a gift by Professor Han Wenling, Peking University. Rat TMEM98 plasmid (pcDNA3.1-TMEM98) was purchased from Shanghai Genema Biotechnology Co. Ltd. HUVEC, AoSMC and A7r5 cells were transfected using Lipofectamine 2000 (Invitrogen, Carlsbad, CA, USA) according to the manufacturer's instructions.

### Statistical analysis

To determine the statistical differences in clinical characteristics among the two groups, we used one-way analysis of variance (ANOVA). Statiscial analysis for *in vitro* experiments and data were expressed as mean ± SD and tested for statistical significance by the 2-tailed Student's t-test using GraphPad Prism 5. All data were representative of at least three independent experiments. Values of *p* < 0.05 were considered statistically significant, where ^*^p < 0.05, ^**^ *p* < 0.01, and ^***^ *p* < 0.001.

## Abbreviations

TMEM98: transmembrane protein 98
AS: atherosclerosis
IL-8: interleukin-8
EC: endothelial cell
VSMC: vascular smooth muscle cell
siRNA: small interfering RNA
RC: regular chow
HFD: high fat diet
BCECF: bis-carboxyethyl-carboxyfluorescein
HGP: human genome project
